# Transcriptomic Analysis of *Prunus domestica* Undergoing Hypersensitive Response to *Plum Pox Virus* Infection

**DOI:** 10.1371/journal.pone.0100477

**Published:** 2014-06-24

**Authors:** Bernardo Rodamilans, David San León, Louisa Mühlberger, Thierry Candresse, Michael Neumüller, Juan Carlos Oliveros, Juan Antonio García

**Affiliations:** 1 Centro Nacional de Biotecnología-CSIC, Campus Universidad Autónoma de Madrid, Madrid, Spain; 2 Center of Life and Food Sciences Weihenstephan, Unit of Fruit Science, Technische Universität München, Freising, Germany; 3 UMR 1332 Biologie du Fruit et Pathologie, CS20032, Villenave d'Ornon, France; University of California, Riverside, United States of America

## Abstract

*Plum pox virus* (PPV) infects *Prunus* trees around the globe, posing serious fruit production problems and causing severe economic losses. One variety of *Prunus domestica*, named ‘Jojo’, develops a hypersensitive response to viral infection. Here we compared infected and non-infected samples using next-generation RNA sequencing to characterize the genetic complexity of the viral population in infected samples and to identify genes involved in development of the resistance response. Analysis of viral reads from the infected samples allowed reconstruction of a PPV-D consensus sequence. *De novo* reconstruction showed a second viral isolate of the PPV-Rec strain. RNA-seq analysis of PPV-infected ‘Jojo’ trees identified 2,234 and 786 unigenes that were significantly up- or downregulated, respectively (false discovery rate; FDR≤0.01). Expression of genes associated with defense was generally enhanced, while expression of those related to photosynthesis was repressed. Of the total of 3,020 differentially expressed unigenes, 154 were characterized as potential resistance genes, 10 of which were included in the NBS-LRR type. Given their possible role in plant defense, we selected 75 additional unigenes as candidates for further study. The combination of next-generation sequencing and a *Prunus* variety that develops a hypersensitive response to PPV infection provided an opportunity to study the factors involved in this plant defense mechanism. Transcriptomic analysis presented an overview of the changes that occur during PPV infection as a whole, and identified candidates suitable for further functional characterization.

## Introduction

*Plum pox virus* (PPV) is the causative agent of sharka, a serious disease that challenges stone-fruit production worldwide [1]–[3]. PPV is a member of the *Potyviridae* family, the largest group of plant viruses [4]–[6]. A single-stranded positive RNA molecule of ∼10 kb forms its genome. At its 5′-end, the RNA is linked to the viral genome-linked protein VPg, and the 3′-end carries a poly-A tail. The genome codes for a large polyprotein and a truncated frameshift product that are processed by three self-encoded proteases into at least 11 proteins [7], [8].

PPV is transmitted by various aphid species in a non-persistent manner [9], [10]. Eight PPV strains have been identified based on their biological, serological and molecular properties as able to infect a wide host range of *Prunus* species [11]–[14]. One strain, PPV-D, regularly infects domestic plum (*Prunus domestica*), causing variable symptoms in leaves and fruits [15]. Only very few *P. domestica* varieties show a hypersensitive response (HR) to PPV infection, e. g., K4-Hybride, Ort×Stan 34 and ‘Jojo’ [16]–[18]; they display necrosis on leaves and bark as well as death of new top sprouts, which stops viral propagation. ‘Jojo’, a descendant of the parent cultivars ‘Ortenauer’ and ‘Stanley’, is the variety with the largest number of PPV isolates analyzed and the largest number of replications; its HR is elicited by all PPV isolates tested (PPV D, M, Rec, EA and W strains) [17]. This makes ‘Jojo’ an attractive candidate for study of the factors involved in this type of resistance.

In plants, proteins encoded by resistance genes (*R* genes) trigger HR through direct or indirect interaction with avirulence proteins, initiating a cascade reaction within the cell. The majority of cloned *R* genes encode nucleotide binding site-leucine-rich repeat proteins (NBS-LRR,) making this family one of the largest, most variable gene families in plants [19]. Several studies have focused on resistance gene analogs in *Prunus* species, the natural host of PPV [20], [21], but to our knowledge, none, addresses the mechanisms of *Prunus* HR to PPV.

Next-generation sequencing, referred to as RNA sequencing (RNA-seq), has proved to be a valuable tool for assessing gene expression differences across the entire transcriptome for a wide range of organisms [22], [23]. Unlike microarrays, these types of analyses can be performed when a genome sequence is unavailable, thus providing information on the biology of non-model organisms [24]–[26]. RNA-seq has proved useful not only for analysis of endogenous genes transcribed in the plant, but also for viral genome reconstruction and recognition; it allows study of the diversity of the infecting viral population, which is relevant for adaptation and survival [27]–[30].

Here we used this technology to compare gene expression between PPV-infected ‘Jojo’ trees at the beginning of an HR response and that of non-infected trees. We performed two studies, one focused on viral reconstruction and heterogeneity analyses, and the other on endogenous plant sequences. The former allowed recognition of an unanticipated isolate of PPV-Rec during the course of the infection, and the latter permitted *de novo* reconstruction of the plant transcriptome and assessment of gene expression changes possibly related to HR in *Prunus*. *In silico* and qPCR tests confirmed the quality and strength of the results.

## Materials and Methods

### Grafting, infection and tissue collection

In year 1, one-year-old *Prunus cerasifera* ‘Myrobalan’ seedlings and *in vitro*-propagated *P. domestica* ‘Wangenheims’ (Weiwa) plants were planted in an insect-proof greenhouse. Half of the plants, for use as rootstock, were inoculated by chip budding in February with a PPV-D isolate present in the Baden region, Germany, using budsticks from PPV-D infected *P. domestica* ‘Katinka’ trees. One year later (year 2), plants were tested for PPV by DASI-ELISA. In mid-May of year 2, two dormant buds of *P. domestica* ‘Jojo’ were chip-grafted onto each plant used as rootstock and began to grow after four weeks. At the first visible symptoms of the hypersensitivity response, young ‘Jojo’ shoots were harvested and frozen immediately in liquid nitrogen. Leaves from 6 or 2 plants, respectively, were used to prepare Jojo-M+PPV and Jojo-W+PPV samples. Young ‘Jojo’ shoots growing on 5 PPV-free rootstocks were sampled simultaneously by the same procedure.

### Total RNA extraction

Frozen tissue was crushed in liquid nitrogen using a mortar and pestle. Total RNA was extracted following the protocol of Carrier et al. [31] modified by Pichaut JP, Labonne G and Dallot S (unpublished; private communication) as follows: 1 g tissue was resuspended in 20 ml preheated buffer 1 [200 mM Tris-HCl pH 8.5, 50 mM EDTA, 500 mM NaCl, 0.5% SDS, 1% PVP40000, 20 mg proteinase K, 1% β-mercaptoethanol] followed by incubation (55°C, 15 min). The sample was aliquoted in 1 ml fractions. KAc (5 M; 300 µl) was added to each tube and incubated on ice (10 min), then centrifuged (10,000 g, 4°C, 10 min). Buffer 2 (1/3 guanidium chloride 7.8 M+2/3 96% ethanol; 450 µL) and 50 µL silica suspension were added to each supernatant and silica was pelleted by centrifugation (1000 g, 2 min, room temperature). Pellets were washed twice with 600 µL buffer 3 (1/3 160 mM KAc, 23 mM Tris-HCl pH 8.0, 0.1 mM EDTA+2/3 170 mL 96% ethanol). Silica was resuspended in 200 µl H_2_O followed by centrifugation (1000 g, 2 min). Supernatants were incubated overnight with 167 µl 8 M LiCl to precipitate RNA. In a final step, RNase-free DNAse treatment (Promega) was performed to remove possible residual DNA.

### RNA-seq and preliminary data processing

Library construction and RNA sequencing were performed by the Beijing Genomics Institute (BGI-Shenzhen, Shenzhen, China). Briefly, from 30 µg total RNA, poly-A+RNA was selected by oligo-dT chromatography, followed by RNA fragmentation. Fragmented mRNA was converted into double-stranded cDNA using random-hexamer primers followed by end repair, 3′ end adenylation and adapter ligation. cDNA fragments were selected by agarose gel extraction and enriched by PCR amplification. The library was loaded onto an Illumina HiSeq 2000 instrument for pair-end sequencing. The average read length of 90 bp was generated as raw data.

Prior to assembly, FastQC software [32] was used to obtain information about the quality of the sequencing data. This information was used for the initial filtering of sequences by the FASTX Toolkit [33], removing the adapters and cleaning low quality sequences (mean Q<20 or more than 20% nucleotides with Q<15) and sequences with unknown nucleotides which could affect later bioinformatic analyses.

### Virus assembly

Virus reconstruction using PPV-D EF569214.1 as reference was done by mapping the sequences from the RNA-seq samples using the Bowtie aligner [34]. Selection of the most frequent nucleotide from the resulting alignments generated the consensus sequence. If the number of aligned reads at a position was zero, the nucleotide was designated ‘N’. A second alignment of the viral reads using the reconstructed viral genome as reference was carried out to obtain the final consensus sequence. *De novo* reconstruction was performed by Oases assembler and the output transcripts were searched against the viral genomes of the NCBI nucleotide database [35] using blastn and blastx. Clustering using CAP3 [36] and CD-HIT [37] followed by extension of the genomic sequence was performed. As in the previous case of viral reconstruction using reference data, the *de novo* rebuilt sequence was used as reference to map the viral reads again using Bowtie aligner.

### Viral heterogeneity analysis of ‘Jojo’ sequences

The population of viral sequences identified in the virus assembly was filtered to avoid possible PCR and sequencing errors. For viral genome alignment, only paired sequences were selected. To avoid insertion and deletion errors, border regions of the sequences were discarded, and only 60 nucleotides of the central region of each sequence was considered. The frequency of each site-specific heterogeneity was calculated as the percentage of mismatches to the consensus sequence of the aligned reads. Since some heterogeneities were artifacts due to the sequencing process, the quality score of each nucleotide read was used to compute the average probability of sequencing error. Assuming that sequencing errors were independent, when the number of alignment mismatches for a certain heterogeneity point was smaller than the expected number of errors (the mean of the binomial distribution B(x;p_i_n_i_) where p_i_ is the average probability of sequencing error in the position i and n_i_ is the coverage in the position i), that point was considered a sequencing error and removed from the analysis. Entropies of the viral populations were calculated as described by Wright et al. [38].

### Analysis of selective pressures

For the analysis of the viral intraspecies, a previous quasispecies formation was done with QuasiRecomb software [39] using nucleotides 892-9568 due to software coverage requirements. Recombination was not allowed to decrease complexity of the analysis. The ratio of non-synonymous-to-synonymous nucleotide-substitution rates (dN/dS) was assessed by Model selection program implemented in Hyphy package [40] and employed for both intra- and interspecies studies. The SLAC [41], a maximum likelyhood method, was used to identify the global synonymous nucleotide-substitution rate (posterior probabilities bigger than 90%).

### Viral diversity analysis of SharCo sequences

To start the analyses, full genome sequences of PPV-D and PPV-Rec isolates stored in the SharCo database [42] were collected. Sequences were aligned using ClustalW [43]. Consensus sequences as well as nucleotide variation data were obtained from the multiple sequence alignment.

### Differential expression

Transcriptome assembly was done with Velvet [44] and Oases programs [45]. Input in both cases was a combination of the four samples and the best output, Velvet + Oases (multi-k), was selected based on the N50 value. Subsequent analyses used the contigs of this assembler. Oases was operated with multi-k option (K = 47, 51, 61, 65, 67, 71, 75) and the option of minimum length of transcript was assigned to 200. Sequences were mapped to the transcripts with BWA [46] and distribution of the expression in the predicted unigene alignments was determined with RSEM [47]. Differential expression analysis was calculated with edgeR [48]. Results were visualized with the FIESTA program [49]. To add functional information to the genes, blastn and blastx were employed using several databases (NCBI EST [50], NCBI nucleotide [35], NCBI protein [51] and PRGdb [52]), and Interproscan to determine significant domains (e-value<10^−5^ in all cases). Blast2GO [53] was used to join all functional annotations and to obtain the enriched GO-terms associated to the differentially expressed unigenes (FDR≤0.005).

### Phylogenetic analyses

We used ClustalW for multiple sequence alignment [43], followed by searching of the best fitted evolutionary model implemented in MEGA5 [54] with ProtTest [55]. A phylogenetic tree was built using the maximum likelihood algorithm with the parameters provided by the best protein model found by MEGA5 [54]. The phylogeny test was done by the bootstrap method. One thousand replicas were used to obtain the percentages of replicate trees in which the associated taxa clustered [56].

### Real-time quantitative PCR (qPCR)

The first cDNA strand was generated from 1 µg total RNA used in the RNA-seq experiment in the presence of oligo(dT)_12-18_ and Superscript III reverse transcriptase (Invitrogen). PCR was performed in triplicates using Fast Universal SYBR Green Master mix (ROX; Roche) in an ABI 7300 Real Time PCR System (Applied Biosystems). Primers were designed using the program Primer3 [57] as shown in Table S5. Gene expression was normalized to the unigenes that matched TEF2 and RPII genes of *P. persica*. Data were calculated by the ΔΔ^CT^ method described by Pfaffl [58] and are shown as the x-fold change in gene expression relative to the control sample.

## Results and Discussion

In this transcriptome analysis, we analyzed four samples by next-generation sequencing. Due to difficulties in direct infection of ‘Jojo’ trees, PPV was inoculated by grafting onto infected rootstocks. Two samples, Jojo-W+PPV and Jojo-M+PPV, correspond to RNA isolated from ‘Jojo’ grafted onto infected Wangenheims (Weiwa) and Myrobalan (Myro) rootstocks, respectively. As control, we used two experimental replicas, Jojo-W1 and Jojo-W2, corresponding to RNA from ‘Jojo’ tissue grafted onto non-infected Weiwa rootstocks. Data from RNA-seq of these four samples were used for *de novo* assembly of contigs using Velvet [44], followed by Oases [45]. A general summary of the data obtained is presented in Table 1.

**Table 1:** Summary of *de novo* sequence assembly using Velvet and Oases.

Group	Read #	Kmer	Unigene #	N50	Unigene # >1 Kb	Max. Len.
Jojo*-W*+PPV	38,907,078	71	33,184	415	906	3,625
Jojo-M+PPV	40,125,506	67	40,655	412	985	3,255
Jojo*-W*1	38,864,360	71	26,400	389	506	3,099
Jojo*-W*2	39,467,930	71	27,820	416	738	3,170
Jojo-M+PPV|Jojo-*W*+PPV	79,032,584	75	35,252	434	1,154	3,153
Jojo-W1|Jojo-W2	78,332,290	73	33,619	445	1,146	3,845
all	157,364,874	75	49,821	464	2,183	3,602

Group	Read #	Kmer	Transcript #/Unigene #	N50	Unigene #>1 Kb	Max. Len.
all	157,364,874	multi	117,919/35,339	1,324	8,200	7,599

The white section of the table shows the data calculated with Velvet; the grey sections indicates the data processed using Oases. **Read#**: total number of reads including viral sequences; **Kmer**: length, in base pairs, of the words being hashed to create the transcripts; **Transcript #**: total number of transcripts; **Unigene #**: total number of unigenes, where a unigene is a hypothetical gene represented by a cluster of similar transcripts thought to be isoforms in the *de novo* transcriptome assembly; **N50**: unigene length such that using equal or longer unigenes produces half the bases of the total unigenes; **Unigene #>1 Kb**: number of unigenes larger than 1 Kb; **Max.Len**.: largest unigene in each group.

Analysis of the viral reads showed notable differences between the two infected samples. Jojo-M+PPV had 150 times fewer PPV reads than Jojo-W+PPV (2,203 and 330,439 reads, respectively), probably caused by experimental factors such as a stronger response to PPV by ‘Jojo’ plants grafted onto Myro, different viral propagation rates between the grafted trees, or other factors difficult to address due to the variable nature of these infection experiments. A few viral reads were found during analysis of the non-infected tissue samples (80 reads in Jojo-W1 and 132 in Jojo-W2), possibly due to contamination during sample preparation or sequencing, but should not affect subsequent analyses (NCBI-SRA study accession: SRP041925).

### Analysis of viral sequences

#### Viral reconstruction

**Figure 1:**
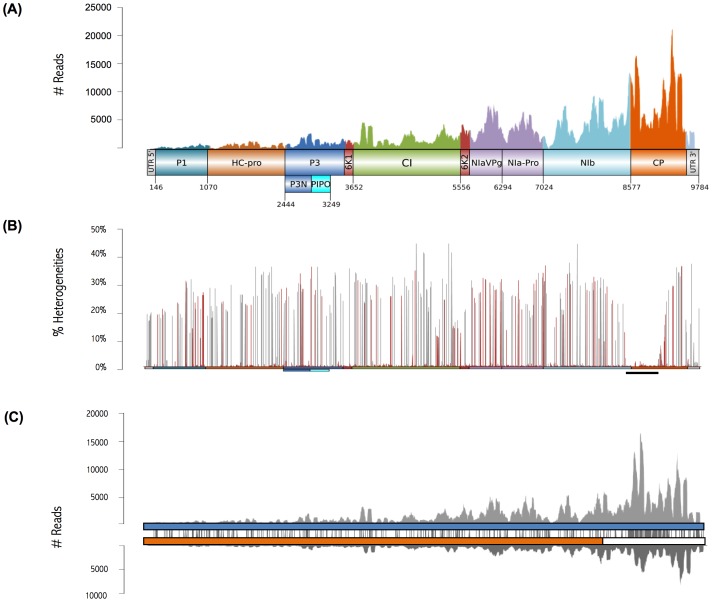
Viral reconstruction using reads from the Jojo-W+PPV sample. (A) Coverage of the viral genome was obtained by the template-based approach. Protein-coding sequences and untranslated regions are labeled and colored. Nucleotide positions at their borders are marked. (B) Heterogeneity analysis. The viral genome is depicted in a colored bar as in (A). Line height indicates the heterogeneity values of the Jojo-W+PPV population relative to the consensus sequence as determined by the template-based approach. Changes that alter the amino acid sequence are in red; region with no significant heterogeneities is indicated by a black line beneath the genome. (C) PPV-D and PPV-Rec sequences obtained after specific clustering of the viral reads. Their corresponding coverages are depicted in grey. Both strains are shown schematically; PPV-D genome (blue), PPV-Rec genome (orange and white). Orange indicates the part of PPV-Rec similar to PPV-D; white indicates the part similar to PPV-M. Nucleotide change distribution between the consensus sequences (grey) is shown in the space between the genomes.

Rootstocks used to infect ‘Jojo’ trees were previously inoculated using budsticks from PPV-D infected *P. domestica* ‘Katinka’ trees. The sequence of this PPV-D isolate was unknown. For viral reconstruction, a viral template (EF569214) was chosen by searching PPV-D in the NCBI database [35]. Alignment of the Jojo-W+PPV reads against this template allowed reconstruction of the near-complete viral genome, using the most abundant nucleotide at each position to build the consensus sequence (Figure 1A). PPV-D isolate FR-65pl from the SharCo database [42] was the most similar viral homologue of PPV-Jojo-W.

For heterogeneity analysis, a position was defined as heterogeneous when occupied by a nucleotide different from that of the consensus sequence. Changes were filtered to avoid base miscalling in the sequencing or amplification processes. A total of 1,261 nucleotide changes were found, and the overall pattern revealed two peculiar features (Figure 1B), the unusually high percentages of heterogeneities in most genomic regions, and the existence of a region at the N-terminal part of the capsid protein (CP) with no marked heterogeneities; this was more striking considering that this region is described as a highly variable genomic region in *Potyviridae* [3].

To test for a possible mistake in the viral reconstruction, we followed a *de novo* approach that also used the assembled contigs from infected samples that matched PPV sequences of the NCBI database [35]. A first analysis retrieved a consensus sequence identical to that obtained using EF569214 as template. Detailed analysis of the results showed the formation of two minor contigs discarded in the first assembly process, which belonged to the recombinant PPV-Rec strain. In this strain, the genome from the 5′ region to the NIb C-terminal region derives from PPV-D, whereas the remainder derives from PPV-M. The two contigs discarded during initial reconstruction belonged to the PPV-M part of PPV-Rec. Six contigs from the PPV-D part of PPV-Rec were sufficiently similar to PPV-D to be included in the first sequence reconstruction, thus contributing to the high genome heterogeneity. In contrast, heterogeneity of the CP N-terminal region, covered by reads from a single isolate, was correspondingly low.

Once the presence of a mixed infection was identified, reads corresponding to one or the other isolate were distributed to reconstruct them and reassess coverage. There were 200,034 reads for the PPV-D isolate and 97,188 reads that matched the PPV-Rec isolate; 17,932 reads could not be assigned to one or the other and were removed from subsequent analyses. New consensus sequences were obtained by *de novo* assembly, as previously detailed. The PPV-D isolate consensus sequence did not vary. The consensus of the PPV-Rec isolate had closest homology to the Slovakian isolate SK-1002ap in the SharCo database [42] (Figure 1C).

The sequence of the virus that infected the Jojo-M sample could only be obtained using the template-based approach, because of the low number of reads available. A total of 25 reads for PPV-Rec were identified from a total of 2,154; this was a lower ratio than in the previous case, but not surprising given the variability of the experiment. The assembled PPV-D sequence had 99.3% sequence similarity at the nucleotide level compared with the PPV-D in the Jojo-W sample (Figure S1A). There were not enough reads to assemble the PPV-Rec sequence in the Jojo-M sample.

#### Heterogeneities in the viral sequences

**Figure 2:**
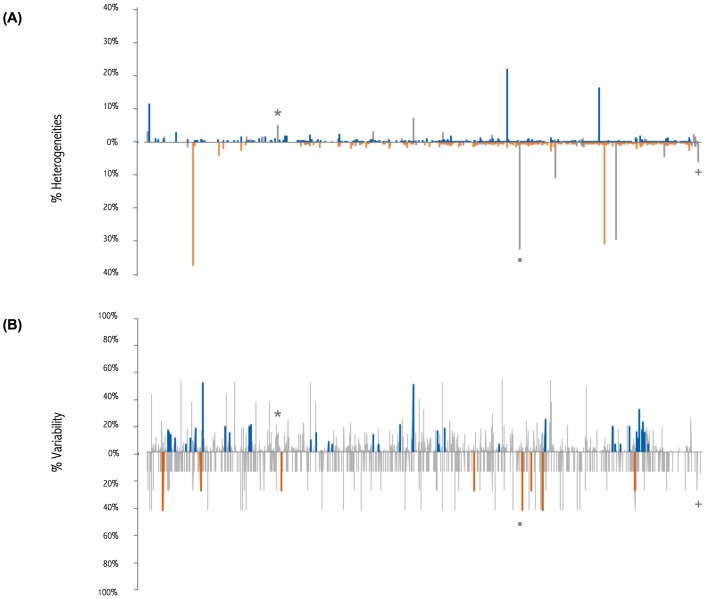
Viral heterogeneities analysis. (A) Heterogeneities in the two major variants in the Jojo-W+PPV viral population. PPV-D (top), with amino acid changes in blue; PPV-Rec (bottom), with amino acid changes in orange. (B) Variability percentages of PPV-D (top) and PPV-Rec isolates (bottom) in the SharCo database [42] that differ from consensus at each polymorphic site. Amino acid changes in PPV-D, blue, and in PPV-Rec, orange. Coincidental changes in (A) and (B) are marked with grey symbols.

To analyze the diversity within the viral populations that infected ‘Jojo’, we studied sequence heterogeneities. Changes were filtered to avoid base miscalling in the sequencing or amplification processes. Changes in both viral sequences in the Jojo-W sample were scattered throughout the viral genome, but increased at the 3′ region (Figure S2A), coinciding with sequence coverage. The entropy of the two populations, computed for the validated sites, showed similar values (PPV-D = 0.0040; PPV-Rec = 0.0039). For PPV-D, we recorded a total of 1,018 positions showing sequence heterogeneity, which comprised ∼10% of the total PPV genome. Of these changes, 665 were non-synonymous. In PPV-Rec, we found half the number of total heterogeneities (584), but the 380 non-synonymous changes maintained a similar non-synonymous-to-synonymous ratio (Figure 2A). There was no notable overlap between the heterogeneities of the two viruses infecting ‘Jojo’ and coincidental changes did not favor non-synonymous selection. These results point out a stochastic origin of the changes. Since chip-budding infection rules out the genetic drift effect of the inoculation process, it can be assumed that stochastic changes are probably favored by the bottleneck effect imposed on viral populations by their invasion of new leaves [59]-[62]. Nonetheless, as the dN/dS analysis shows, a slight purifying selection can be considered in both cases (PPV-D = 0.67, PPV-Rec = 0.80) and this is in agreement with results from a previous experiment in *P. domestica* infected with PPV-D, PPV-Rec and PPV-M, in which viruses displayed weak negative selective pressures [63]. The idea of viral seclusion [59], [64] cannot be addressed here, since the sample analyzed is a pooled sample from different leaves and trees.

Sequences of full PPV-D (84 sequences) and PPV-Rec (8 sequences) isolates in the SharCo database [42] were used to build two consensus sequences and address inter-isolate variability. In both cases, changes were evenly distributed throughout the genome (Figure S2B). PPV-D and PPV-Rec showed 109 common variable sites and, in contrast to the intra-isolate heterogeneity of the ‘Jojo’ samples, changes at nucleotide three of codon triplets were more frequent than in the other two positions (27, 34 and 159 for first, second and third nucleotides, respectively). This is expected for an analysis of inter-isolate variability performed on consensus sequences that have undergone evolutionary pressure.

We found few high percentage heterogeneities (>5%) in viral sequences in the Jojo-W; there were four in PPV-D, three of which were non-synonymous, and five in PPV-Rec (two non-synonymous). None of the changes were coincidental between strains. The non-coding change of ‘Jojo’ PPV-D (position 2340) appeared in the SharCo PPV-D variability, and two non-coding changes of ‘Jojo’ PPV-Rec (positions 6597 and 9721) were also present in the PPV-Rec variability in SharCo. None of the amino acid changes in the ‘Jojo’ viral sequences was found in the SharCo reconstructed consensus sequences (Figure 2B). Given the small number of related changes, it is difficult to address their statistical significance, but the fact that they appear at the nucleotide level supports a random origin.

We analyzed the heterogeneities in the Jojo-M+PPV sample for the PPV-D reconstructed sequence as for the Jojo-W+PPV. Distribution of changes was not homogeneous,and heterogeneities were again more numerous at the 3′ end of the genome (Figure S1B). There was no notable overlap between changes in this sequence and those in the other infected sample. The pattern was nonetheless similar to that observed for the inter-isolate analyses, with changes in the third nucleotide position favored over changes in the other two (6, 9 and 52 for first, second and third nucleotides, respectively).

### Analysis of the plum tree transcribed sequences

#### General overview

**Figure 3:**
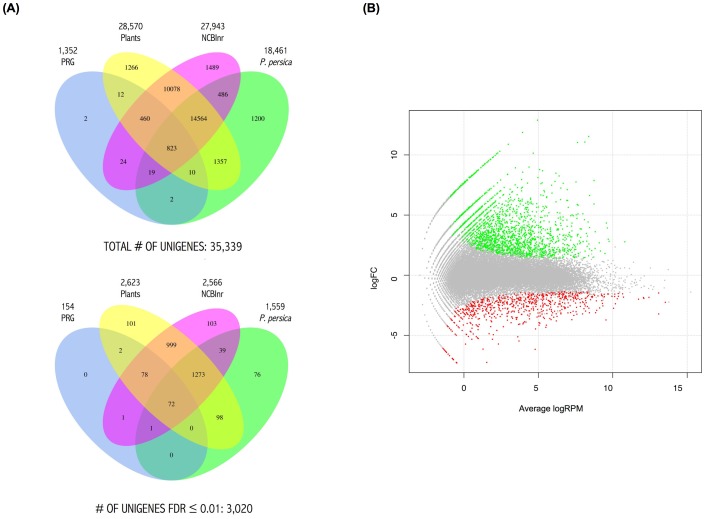
Differential expression of the ‘Jojo’ sample unigenes. (A) Venn diagrams crossing all unigenes in the four databases as indicated (top) and crossing differentially expressed unigenes in the four databases as indicated (bottom). (B) The smear plot represents the tagwise log FC (fold changes of unigene reads in infected versus non-infected samples) against log of average number of unigene reads in the sample set, per million total reads (RPM) of all unigenes. Green and red dots represent up- and downregulated unigenes, respectively, with an FDR≤0.01.

The combined reads from all four samples were used to assemble 117,919 transcripts that corresponded to 35,339 unigenes (Table 1). Blast search of these unigenes was performed against four databases, Plant Resistance Genes database (PRGdb) [52], *Prunus persica* EST [50], plants EST [50] and nr/nt NCBI [35], [51]. To analyze the number of mapped reads against the unigene sequences, the BWA [46] aligner was used with default parameters. Statistical analysis was performed using EdgeR [48], a bioconductor package (www.bioconductor.org) for RNA-seq. Total data were filtered to identify the differentially expressed unigenes with statistical significance, using a threshold of false discovery rate (FDR) equal to or less than 0.01. A visual summary of the results obtained by comparing the four databases can be seen in Figure 3A. A total of 3,547 unigenes did not match any sequence in the databases used.

**Figure 4:**
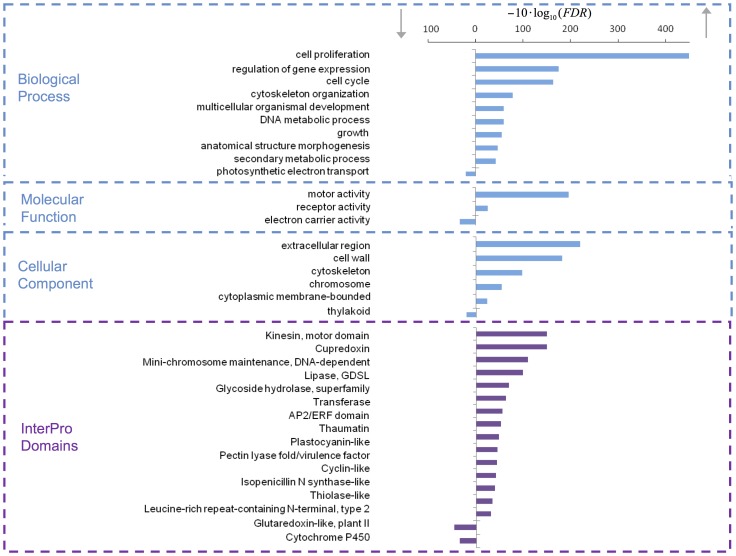
Gene Ontology (GO) enrichment analysis of up- and downregulated genes. Blue boxes separate the different areas of GO-Term distribution. The purple box delimits the Interpro domain distribution. Values are inversely proportional to the False Discovery Rate (FDR) as calculated by Blast2GO. Bar plots corresponding to Interpro functional domains are simplified, joining related functions, following the Interpro relationships inheritance data.

The 35,339 unigenes are depicted on a smear plot (Figure 3B). A total of 3,020 unigenes were found to be differentially expressed in infected versus non-infected samples; 2,234 genes were upregulated and 786 genes were downregulated (Table S1). Homologs for 2,843 of these unigenes were detected in the PRGdb, *P. persica* EST, plants EST or nr/nt NCBI databases. To describe the main pathways modified in ‘Jojo’ plants during PPV infection, we studied the unigenes by Gene Ontology assignment, or GO-term (hypergeometric test, FDR<0.005) (Figure 4). Cell proliferation and motor activity were highlighted among the enriched terms in biological process and molecular function categories, respectively. Genes associated with cellular components such as extracellular region, cell wall, cytoskeleton or chromosome were upregulated; photosynthetic electron transport, electron carrier activity and thylakoid were downregulated terms of the three categories (Figure 4). Analyses of the unigene coding proteins by protein domain search using the Interpro database [65] retrieved groups of proteins similar to those obtained by GO-term search. Thus, proteins involved in chromosome maintenance, motor and kinesin activities, hydrolases or cyclin-like proteins were among those upregulated; glutaredoxin-like proteins and cytochrome P450 were downregulated. Interpro allowed detection of proteins with other domains that might be related to defense response such as thaumatins [66], lipases [67] or proteins with leucine-rich repeats (Figure 4).

A general examination of related studies [68]–[72] underscores two shared features, repression of photosynthetic function in necrotic tissue and enhancement of defense gene expression; these common features are also observed in the present analysis. A search for more specific terms, such as cell wall or metabolism, retrieved contrasting results, which is unsurprising given the broad differences between studies. The overall view presented in our study resembles those shown previously and fits the general idea of a cell experiencing pathogenic stress and undergoing profound changes, with modifications in protein activity, cascade pathways, trafficking and morphology.

#### One hundred fifty-four differentially expressed unigenes are found in the PRGdb database

To determine more directly the elements involved in the hypersensitive reaction, we studied the genes implicated in plant defense and resistance. Of the total number of unigenes, 1,094 matched entries in the PRGdb; of these, 154 were expressed differentially after PPV infection, 126 up- and 28 downregulated (Table S2). More than 50% of these unigenes corresponded to proteins with suspected kinase activity, and 13%, mainly among those upregulated, coded for LRR receptors. Moreover, a small percentage of resistance-related upregulated unigenes (4%) encoded proteins with presumed nucleotide binding activity. Overall, ∼20% of the unigenes that matched PRGdb sequences showed miscellaneous functions and 10% of the total had no defined function.

**Figure 5:**
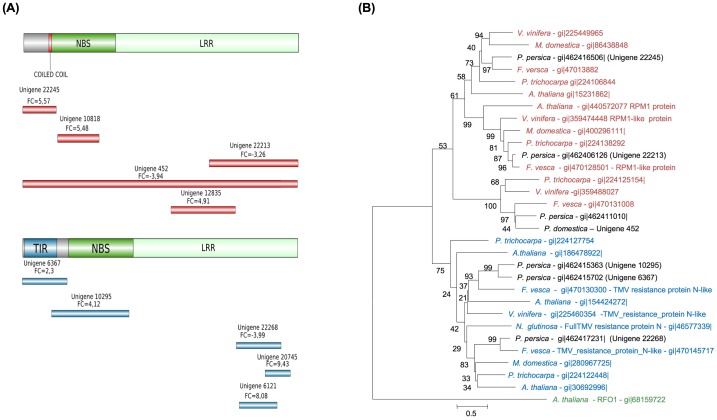
NBS-LRR-type ‘Jojo’ unigenes differentially expressed as a result of PPV infection. (A) Scheme showing unigenes defined as CC-type (red) or TIR-type (blue) according to their sequences or to those of closest relatives identified by Blast analysis. Fold changes (FC) are depicted above each unigene. (B) Phylogenetic tree using the translated sequence of ‘Jojo’ unigene 452 and the closest homologues in *P. persica* (80% or higher) of six ‘Jojo’ unigenes. NBS-LRR translated genes from *F. vesca, P. trichocarpa, V. vinifera*, *M. domestica*, and *A. thaliana* were selected from the NCBI database based on sequence similarity. The *N* protein of *N. glutinosa* was also included in the tree. Color code as in (A) with the addition of green for the outsider protein used to root the tree. Curated *R* proteins are marked next to the gi number. Bootstrapping numbers are located next to each branch.

Due to the importance of NBS-LRR genes in resistance to viral infections [73], we focused specifically on these types of genes. In general, after pathogen detection, *R* genes unleash a cascade mechanism within the cell that results in HR and systemic acquired resistance signaling. For the plant to provide an organized reaction, *R* gene expression must be tightly controlled during pathogen attack; nonetheless, the mechanisms responsible for this regulation remain a mystery. There are reports of *R* gene upregulation following infection, indicating a dosage effect [74]–[76], whereas others describe *R* genes for which expression levels remained unchanged during HR, suggesting a distinct activation mechanism [77], [78]. In our RNA-seq analysis, we identified 314 unigenes as members of the NBS-LRR family, of which seven were upregulated. Size and distribution of the reconstructed unigenes is shown in Figure 5A. Four of the upregulated unigenes appeared to code for *Tobacco mosaic virus*-resistance N-like proteins, with a Toll interleukin-like receptor at the N-terminal part (TIR-NBS-LRR). The other three encoded proteins with a coil-coiled region at the N terminus (CC-NBS-LRR), with no specific gene similarity.

Three NBS-LRR-related unigenes were downregulated (Figure 5A). One of these belongs to the TIR-NBS-LRR family, whereas the other two are of the CC-NBS-LRR type. To our knowledge, there are no reports in which *R* genes described to be involved in HR underwent downregulation during infection. The closest scenario was described for *Arabidopsis thaliana* plants in which the RPM1 protein, which confers resistance to *Pseudomonas syringae*, was degraded as HR progressed [79]. This downregulation occurs at the protein level, but the final result is analogous to a decrease in mRNA accumulation. One of the CC-NBS-LRR unigenes shown here to be downregulated, unigene 22,213, resembles the *RPM1* gene, suggesting a similar mode of action.

Six of the differentially expressed unigenes can be assigned to homologous *P. persica* genes (>80% identity) and their translated proteins can be used to build a phylogenetic tree with their closest NBS-LRR relatives from other plant species whose genome has been completely sequenced. *Fragaria vesca* and *Malus domestica* were selected as other *Rosaceae* species, *Populus trichocarpa* and *Vitis vinifera* as representatives of woody plants, and *A. thaliana* as the most-used model plant species (Figure 5B). Since the complete unigene 452 transcript was reconstructed, both its translated protein and its *P. persica* homolog were included in the phylogenetic analysis. The N protein from *Nicotiana glutinosa* was also included, and a resistance protein from the PRGdb that did not belong to the NBS-LRR type was used as an outgroup to root the tree. In all cases, the *F. vesca* R protein was the closest relative to the translated *P. persica*-related unigenes, and the topology of the tree suggested that they were *bona fide* homologues. The tree showed separate clusters of N- and RPM1-like proteins, including the corresponding *Prunus* homologues, which suggests high conservation of these proteins. In the other cases, the R protein homologues identified did not show a defined function.

#### Genes not in the PRGdb that might be involved in plant defense

Among the 3,020 unigenes differentially expressed during PPV infection, some were not described as *R* genes, but might be involved in plant defense and HR. To perform a detailed gene-to-gene analysis based on literature findings, we first narrowed the unigene population. The unigenes selected were those absent from the PRGdb and with an absolute FC>20 and an FDR≤0.01. This reduced the list to 703 unigenes, a manageable number for a comprehensive study; of these, 75 were chosen as possibly involved in plant response to PPV (Table S3). The list included a wide range of genes coding for proteins as diverse as histones [80], aspartic proteases [81] or polyphenol oxidases [82] and define a clear starting point for future research on plant defense.

### Data quality control: *in silico* and experimental tests

#### *In silico* analysis

Read quality was assessed following well-established parameters in RNA-seq protocols (see Methods). Data were further validated by the statistical parameters of the programs used. As an additional checkpoint, we performed a so-called fold change test. Reconstructed unigenes do not necessarily cover the entire sequence of a gene, and two or more unigenes can correspond to the same gene; these sequences would present similar up- or downregulation expression patterns. For simplicity, this test was carried out only with the differentially expressed unigenes in PRGdb [52]. In this database, seven genes were matched by more than one unigene, and fold changes were consistent in all cases (Table S4). To illustrate this test, two representative examples in which several unigenes were part of a single gene are detailed graphically (Figure S3).

#### Experimental analysis

Five unigenes with distinct expression patterns were selected for confirmatory analysis by qPCR. Due to the lack of data for the domestic plum, two reference genes were selected, TEF2 and RPII, based on the work of Tong et al. [83] in peach trees. Average and individual results are shown in Table 2, together with the results for these five genes in RNA-seq analysis. The remarkable level of reproducibility of the data obtained using these two approaches validates the consistency of this transcriptomic study.

**Table 2:** Comparison of qPCR and RNA-seq data.

UniGene	qPCR					RNA-seq				
	FC ref. unigene 491 (TEF2)		FC ref. unigene 18138 (RPII)		Average FC	# reads				Normalized FC
	W+PPV	M+PPV	W+PPV	M+PPV		W1	W2	W+PPV	M+PPV	
9,718	5.9	9.6	8.2	18.4	10.5±5.5	19	11	300	825	30.1
9,603	13.7	9.9	19.2	19.1	15.5±4.5	13	4	139	162	14.1
6,367	1.9	2.7	2.7	5.2	3.1±1.4	273	279	501	1,041	2.3
9,076	−1.4	−3.1	−1.0	−1.6	−1.7±0.9	787	718	780	574	−1.4
5,222	−16.5	−106.4	−11.7	−55.4	−47.5±43.8	1,249	1,157	110	31	−21.3

Efficiencies of the primers: 9,718 = 1.9; 9,603 = 1.92; 6,367 = 2.07; 9,076 = 1.95; 5,222 = 2.16; 491 = 1.95; 18,138 = 1.98. Average FC: average fold change of the qPCR values. Normalized FC: normalized fold change based on total number of endogenous reads of each sample.

## Conclusions

RNA-seq has proven to be a valuable technique to study gene expression profiles in model and non-model organisms. To our knowledge, this is the first report that attempts expression characterization of *Prunus* trees developing an HR response to PPV infection. This study allowed not only reconstruction of the PPV-D viral genome consensus sequence, but also analysis of the genetic complexity of the viral population, which led to identification of a second, previously undetected major viral strain, PPV-Rec. We identified 3,020 unigenes that are differentially expressed during infection, 154 of which are related to genes implicated in defense responses. Of these unigenes, 10 were of the NBS-LRR type. Closer analysis of the data identified additional candidate genes that show large variations in their mRNA levels and might be involved in viral resistance, establishing the basis for further functional analyses.

## Supporting Information

Figure S1 **Viral reconstruction using reads found in the Jojo-M+PPV sample.** (A) Coverage of the viral genome. Protein-coding sequences are labeled and colored. The nucleotide positions at their borders are marked. (B) Heterogeneity analysis. The viral genome is depicted using a colored bar as in (A). Line height indicates the heterogeneity values of the Jojo-M PPV population (above the genome) in the indicated position. Amino acid differences are depicted in red. Below the genome, grey and red lines represent silent and non-silent differences, respectively, relative to Jojo-W+PPV consensus sequence. The green line shows an amino acid change in PIPO.
(TIF)

Figure S2 **Intra- and inter-isolate heterogeneities analysis.** (A) Intra-isolate heterogeneities distribution in PPV-D (blue) and PPV-Rec (orange) from the Jojo-W+PPV sample. Heterogeneities are grouped by 490 nucleotides. Grey lines between the two strains mark changes common to both. (B) Inter-isolate variability distribution in PPV-D (blue) and PPV-Rec (orange) from the SharCo database [42]. Polymorphic sites are grouped by 490 nucleotides. Grey lines between the two strains mark changes common to both.
(TIF)

Figure S3 **Parallel fold change test.** Schemes of two genes from the PRGdb [52] that are matched by several differentially expressed ‘Jojo’ unigenes. Green (W1 and W2) and red (W and M) boxes show the numbers of reads corresponding to non-infected and infected samples, respectively.
(TIF)

Table S1 **Differentially expressed unigenes.** The raw number of reads found on each sample is given in columns Jojo-W1, Jojo-W2, Jojo-W+PPV and Jojo-M+PPV. Fold changes between infected and non-infected samples and FDR values are detailed in columns FC and FDR, respectively. Column labeled Description expands on the details of each unigene, including length in nucleotides.
(XLS)

Table S2 **Differentially expressed unigenes found in PRGdb.** The raw number of reads found on each sample is given in columns Jojo-W1, Jojo-W2, Jojo-W+PPV and Jojo-M+PPV. Fold changes between infected and non-infected samples and FDR values are detailed in columns FC and FDR, respectively. Column labeled Description expands on the details of each unigene, including length in nucleotides.
(XLS)

Table S3 **Genes possibly involved in plant defense not included in the PRGdb.** Similarity to a known protein is given in the Homology column. The raw number of reads found on each sample is given in columns Jojo-W1, Jojo-W2, Jojo-W+PPV and Jojo-M+PPV. Fold changes between infected and non-infected samples and FDR values are detailed in columns FC and FDR, respectively. Column labeled Description expands on the details of each unigene, including length in nucleotides.
(XLS)

Table S4 **List of genes from PRGdb matched by more than one ‘Jojo’ unigene.** Reference numbers of the matched genes are shown in the NCBI Match column. The raw number of reads found on each sample is shown in columns Jojo-W1, Jojo-W2, Jojo-W+PPV and Jojo-M+PPV. Fold changes between infected and non-infected samples is given in column FC. Column labeled Description expands on the details of each gene.
(XLS)

Table S5 **Primers used in qPCR experiment.**
(DOC)
