# Supplementary figures and images for: Transcriptomic Analysis of Prunus domestica Undergoing Hypersensitive Response to Plum Pox Virus Infection
Source: PLoS One. 2014 Jun 24;9(6):e100477. doi: 10.1371/journal.pone.0100477 (PMC4069073; doi:10.1371/journal.pone.0100477)

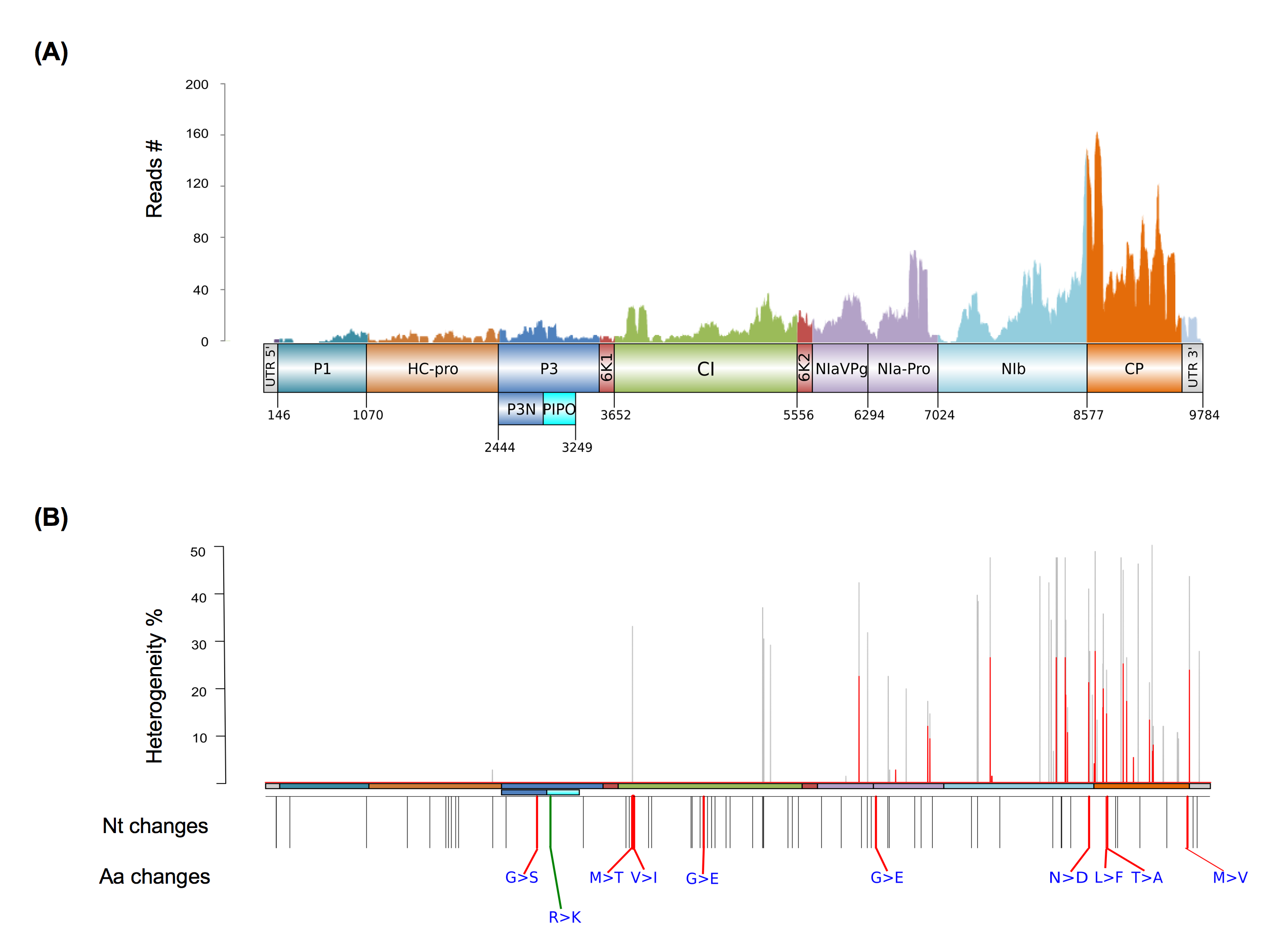

Supplement: Figure S1 — Viral reconstruction using reads found in the Jojo-M+PPV sample. (A) Coverage of the viral genome. Protein-coding sequences are labeled and colored. The nucleotide positions at their borders are marked. (B) Heterogeneity analysis. The viral genome is depicted using a colored bar as in (A). Line height indicates the heterogeneity values of the Jojo-M PPV population (above the genome) in the indicated position. Amino acid differences are depicted in red. Below the genome, grey and red lines represent silent and non-silent differences, respectively, relative to Jojo-W+PPV consensus sequence. The green line shows an amino acid change in PIPO. (TIF) [file pone.0100477.s001.tif]

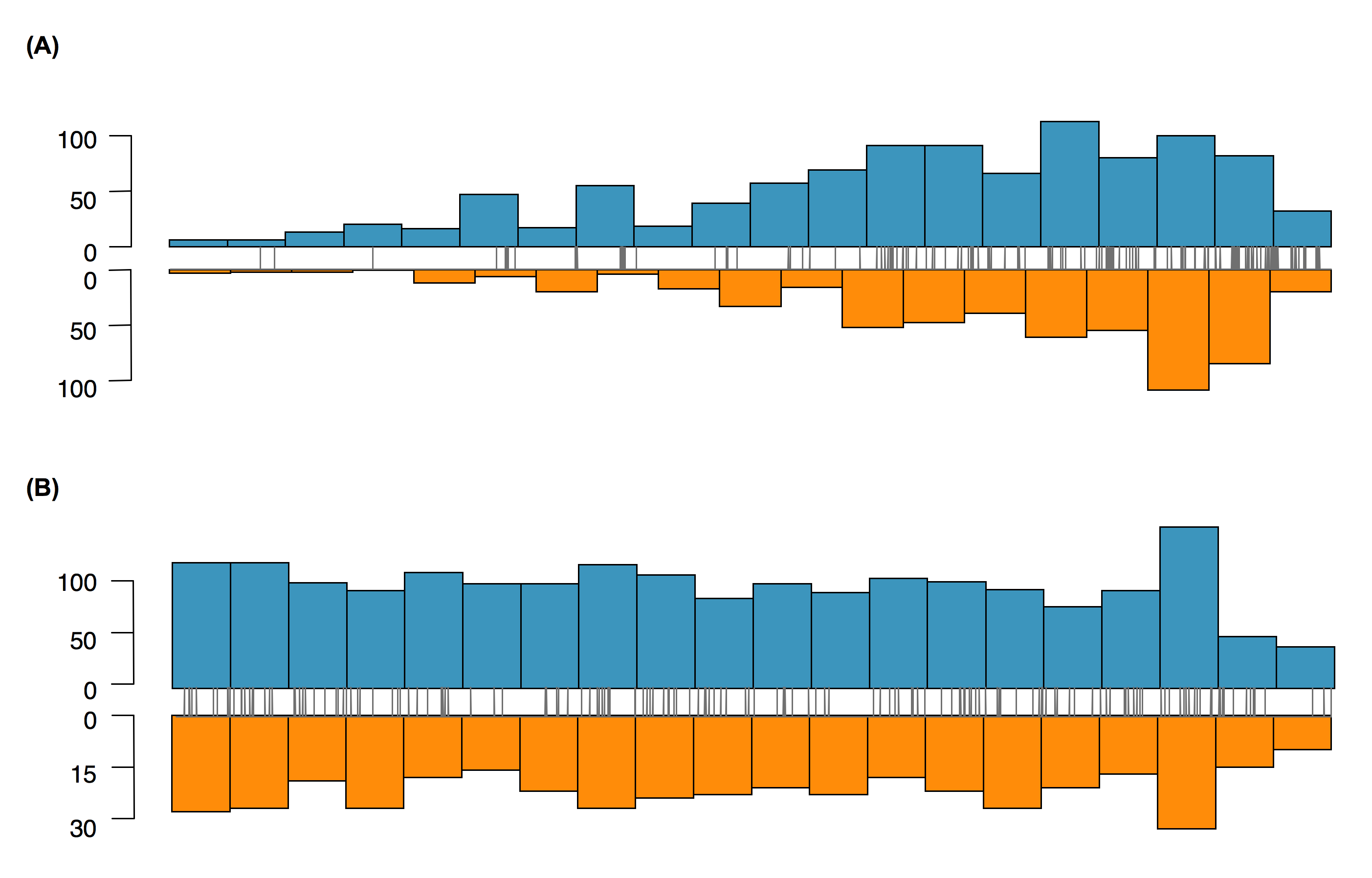

Supplement: Figure S2 — Intra- and inter-isolate heterogeneities analysis. (A) Intra-isolate heterogeneities distribution in PPV-D (blue) and PPV-Rec (orange) from the Jojo-W+PPV sample. Heterogeneities are grouped by 490 nucleotides. Grey lines between the two strains mark changes common to both. (B) Inter-isolate variability distribution in PPV-D (blue) and PPV-Rec (orange) from the SharCo database [42]. Polymorphic sites are grouped by 490 nucleotides. Grey lines between the two strains mark changes common to both. (TIF) [file pone.0100477.s002.tif]

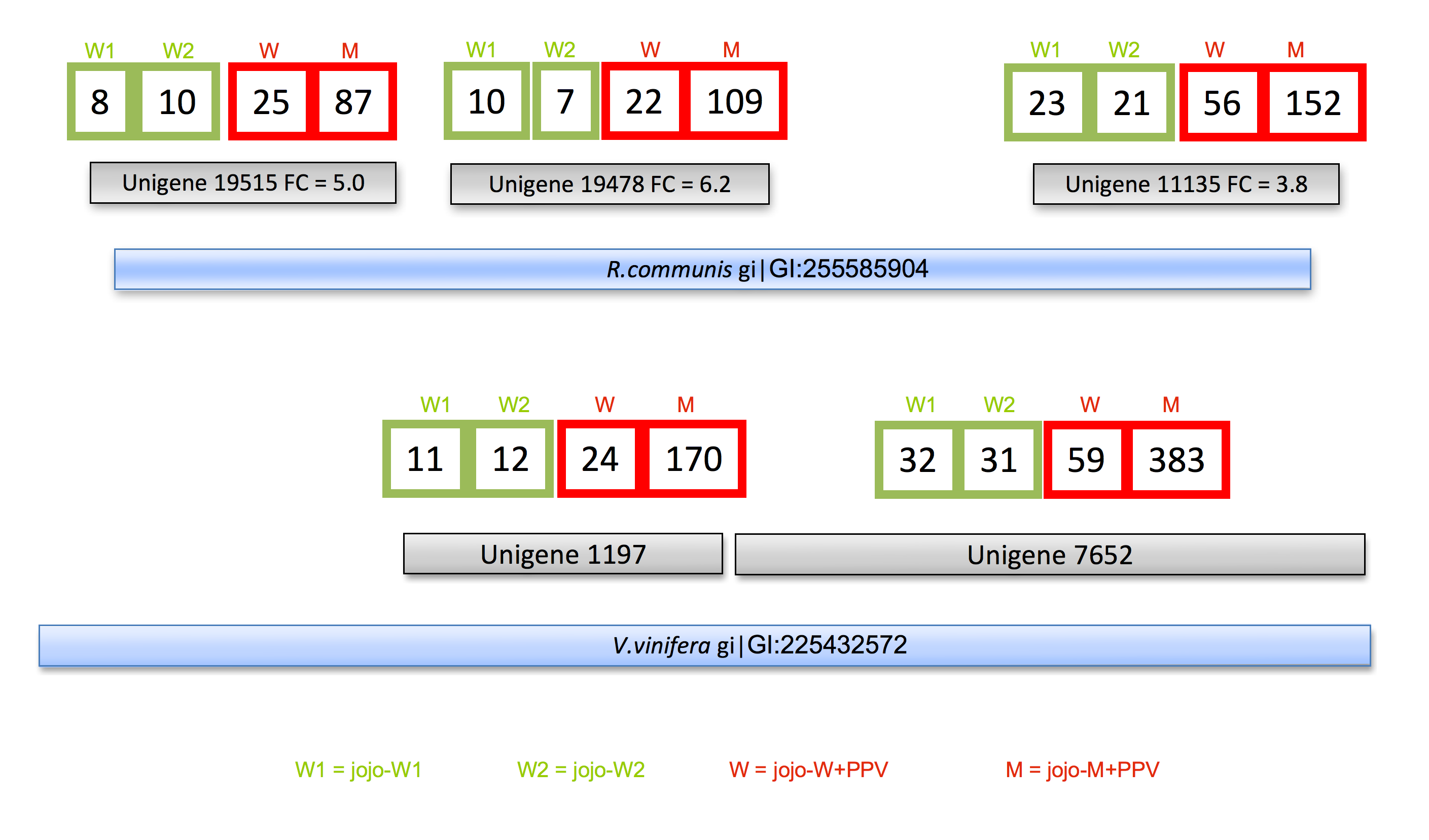

Supplement: Figure S3 — Parallel fold change test. Schemes of two genes from the PRGdb [52] that are matched by several differentially expressed ‘Jojo’ unigenes. Green (W1 and W2) and red (W and M) boxes show the numbers of reads corresponding to non-infected and infected samples, respectively. (TIF) [file pone.0100477.s003.tif]
