# Supplementary material for: Transcriptomic Analysis of Prunus domestica Undergoing Hypersensitive Response to Plum Pox Virus Infection
Source: PLoS One. 2014 Jun 24;9(6):e100477. doi: 10.1371/journal.pone.0100477 (PMC4069073; doi:10.1371/journal.pone.0100477)
Supplement: Table S5 — Primers used in qPCR experiment. (DOC) [file pone.0100477.s008.doc]

**Table S5. Primers used in qPCR experiment**

| **Unigene** | **Primer 5´->3´** | **Fragment size** |
| --- | --- | --- |
| **9718** | F- AGCAAATGGAGCTAGCCGTA | 183 |
|  | R- TGTGGCACAGGAGCTATGAG |  |
| **9603** | F- GGGGTGATTGGAGTTATTGC | 214 |
|  | R- CAGCGCTACCTTTAGCCAAC |  |
| **6367** | F- CTTTGAGCTTCTCCATACTTC | 151 |
|  | R-TGATCCGTAAAATTGGTGCAT |  |
| **9076** | F- CCCAAAACCTTTGCCAATAA | 180 |
|  | R- AATCGCTCAGACGCTTCCTA |  |
| **5222** | F- GGATGTAAATGGCCATCAGG | 207 |
|  | R- TCAGCAGTAGCACCACCAAG |  |
| **TEF 2** | F- TAACCCTCTGGAATGCTTGG | 172 |
|  | R- GCACTTGTGGTGGTTGATTG |  |
| **RPII** | F- GGTTGTGAATGGGATGGAAC | 212 |
|  | R- TCACTACGGGCCCAACTATC |  |
